# THE RELATIONSHIP BETWEEN ORGANIZATIONAL CULTURE AND FINANCIAL PERFORMANCE AMONG HIGH-MEDICAID NURSING HOMES

**DOI:** 10.1093/geroni/igz038.2570

**Published:** 2019-11-08

**Authors:** Robert Weech-Maldonado, Akbar Ghiasi, Ganisher K Davlyatov, Justin C Lord, Jane Banaszak-Holl

**Affiliations:** 1 University of Alabama at Birmingham, Birmingham, Alabama, United States; 2 Louisiana State University at Shreveport, Shreveport, United States; 3 Monash University, Melbourne, Victoria, Australia

## Abstract

This study examines the relationship between organizational culture and financial performance of high Medicaid census (70% or higher) nursing homes (NHs). Based on the Competing Values Framework, there are four types of organizational culture: clan culture (friendly working environment); adhocracy culture (dynamic/creative working environment); market culture (results-based organization); and hierarchy culture (formalized/structured work environment). This study used facility survey data from approximately 324 nursing home administrators (30% response rate) from 2017- 2018, merged with secondary data from LTCFocus, Area Health Resource File, and Medicare Cost Reports. The dependent variable consisted of the operating margin, while the independent variable comprised type of organizational culture. Control variables were organizational (ownership, chain affiliation, size, occupancy rate, and payer mix), and county-level factors (Medicare Advantage penetration, income, education, unemployment rate, poverty, and competition). Multivariable regression was used to model the relationship between organizational culture type and financial performance. Regression results show that compared to a market culture, a hierarchy culture was associated with an 11.8 % lower operating margin, a clan culture with a 10.6% lower operating margin, and a non-dominant culture with 11.4% lower operating margin. Organizational culture is associated with financial performance among high Medicaid facilities, with market cultures outperforming other organizational cultures. Given increasing competition in the nursing home market and declining resources for high Medicaid nursing homes, facilities with a more external orientation and focus on results may be able to perform better financially. Future research should examine the effect of organizational culture on quality of care.

